# Supplementary material for: High-fidelity DNA replication in Mycobacterium tuberculosis relies on a trinuclear zinc center
Source: Nat Commun. 2017 Oct 11;8:855. doi: 10.1038/s41467-017-00886-w (PMC5636811; doi:10.1038/s41467-017-00886-w)
Supplement: Supplementary file 1 — Supplementary Information [file 41467_2017_886_MOESM1_ESM.pdf]

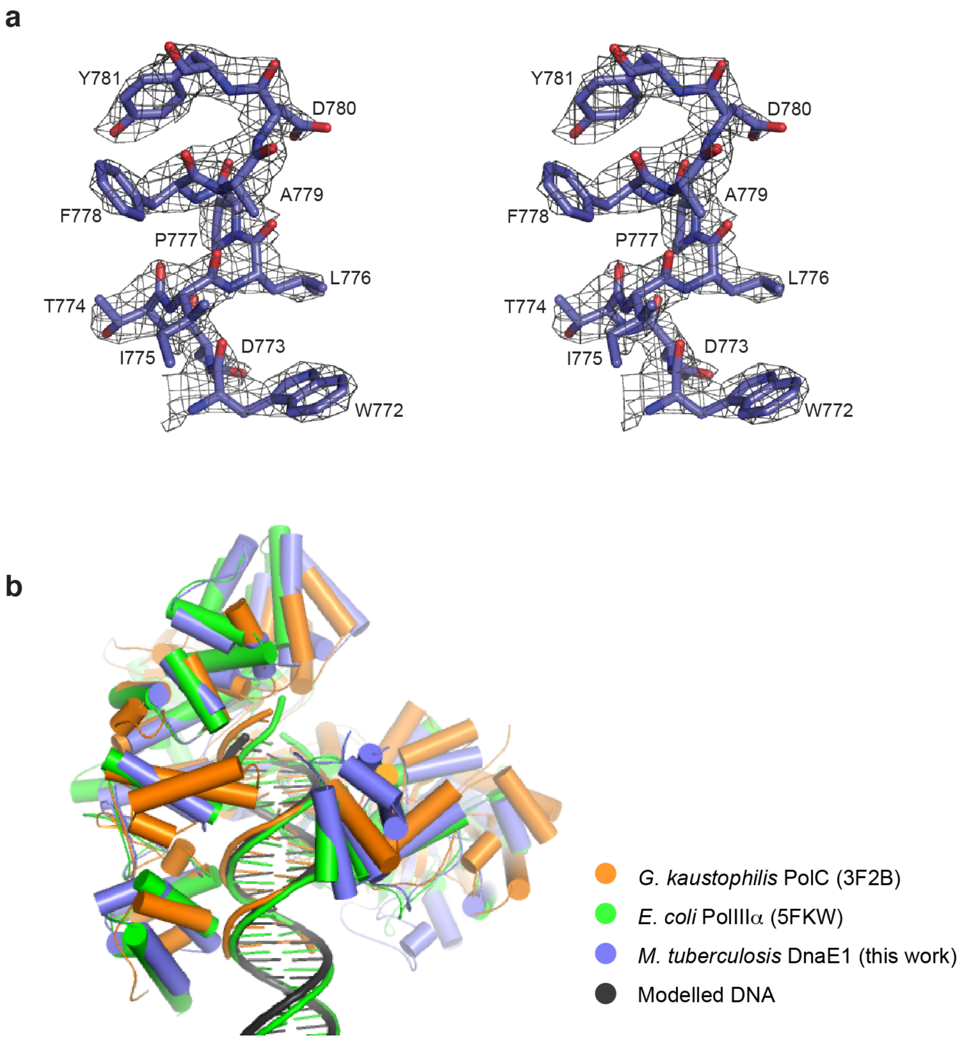

Supplementary Figure 1 | Stereo view of electron density and modeling of a DNA substrate into the crystal structure of Mtb DnaE1.

(**a**) Stereo view of a 2mFo-DFc electron density map contoured at 2.1σ (**b**) Modeling of DNA binding. The *Geobacillus kaustophilis* PolC-DNA complex[1] and *E. coli* PolIIIα-DNA complex[2] were superimposed onto the structure of Mtb DnaE1 using the palm and fingers domain (residues 307-933) as a guide. Next, a B-form dsDNA model was manually aligned with the DNA from PolC and PolIIIα.

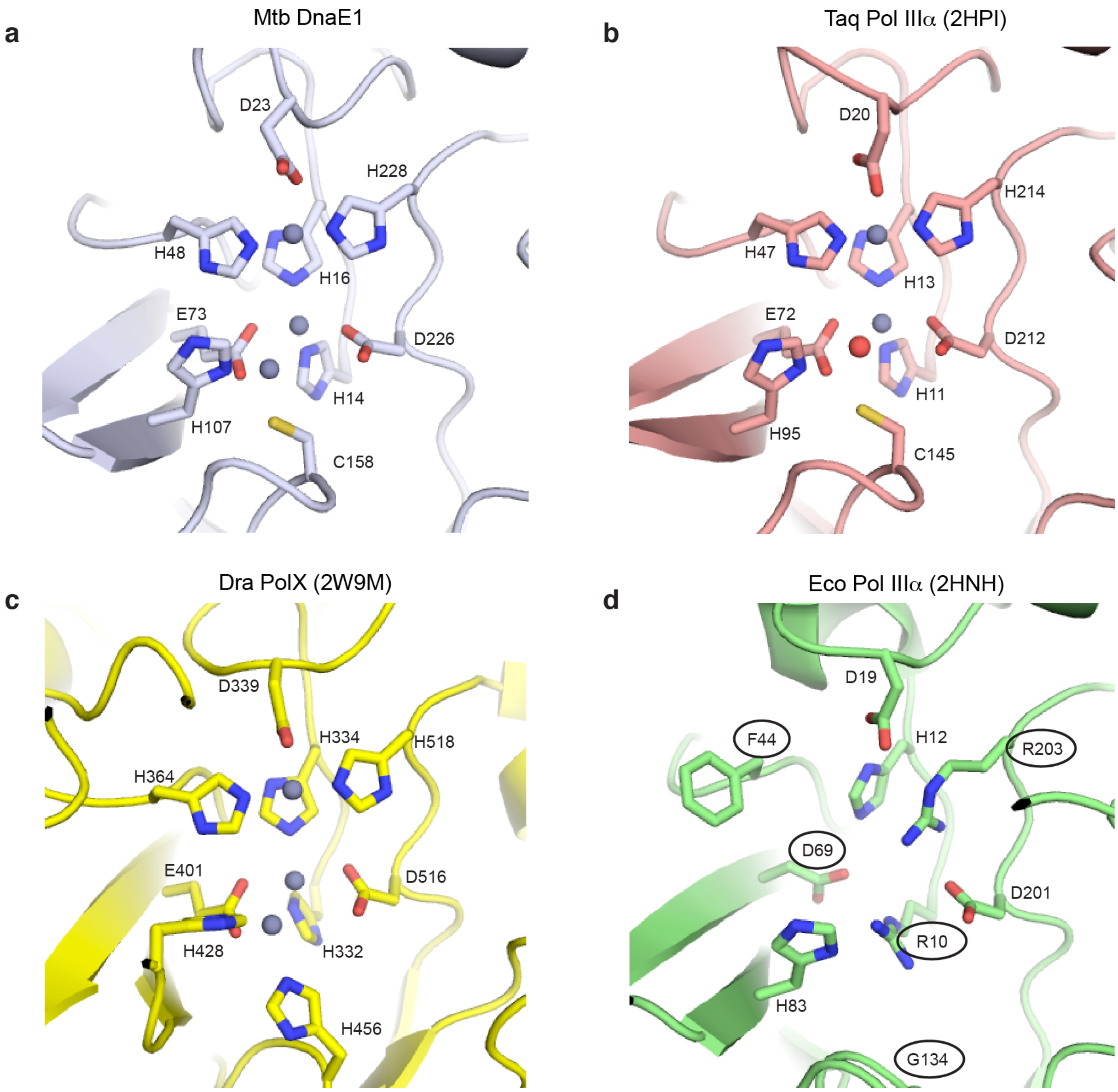

Supplementary Figure 2 | Conserved features of the PHP-exonuclease active site. (**a**) PHP active site of Mtb DnaE1. The metal binding residues shown in stick, and Zn ions in grey spheres (**b**) PHP active site of Taq PolIIIα[3]. Note that one of the metal ions is modeled as a water molecule (shown in red sphere). (**c**) PHP active site from *Deinococcus radiodurans* PolX[4] (**d**) PHP active site from *E. coli* PolIIIα[5]. The five residues that deviate from the consensus sequences are indicated with circles. Note that no metals are bound in the active site.

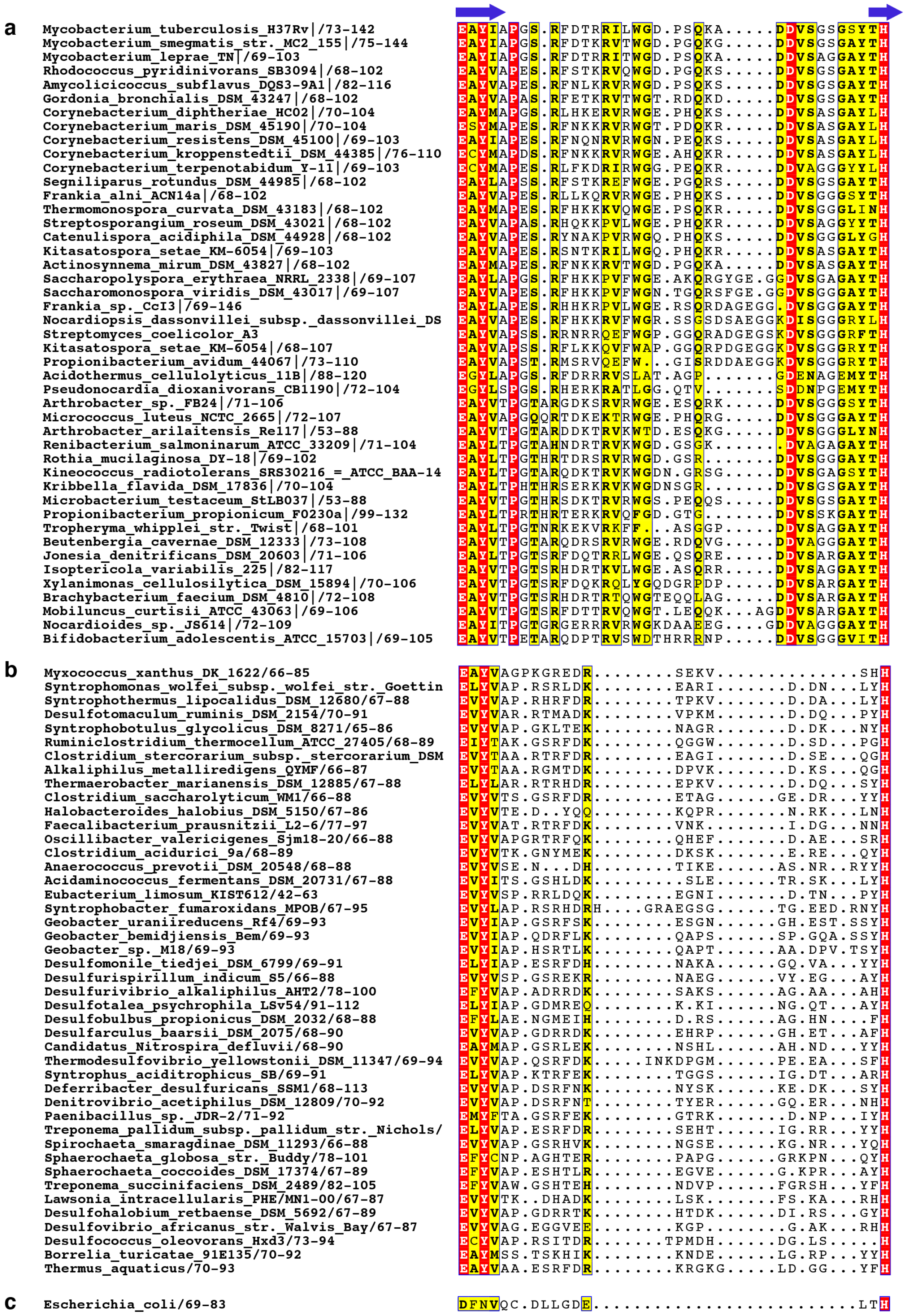

Supplementary Figure 3 | Sequence conservation of the long loop in the PHP-exonuclease. Alignment of the long loop in the PHP-exonuclease, equivalent to glutamate 73 to histidine 107 from Mtb DnaE1 (see Sup. Fig. 2). Blue arrows on top indicate the β-strands flanking the loop. (**a**) 45 sequences from Actinobacteria (**b**) 45 sequences from δ-proteobacteria and Firmicutes (**c**) The equivalent sequence from *E. coli* PolIIIα, which is an inactive PHP-exonuclease.

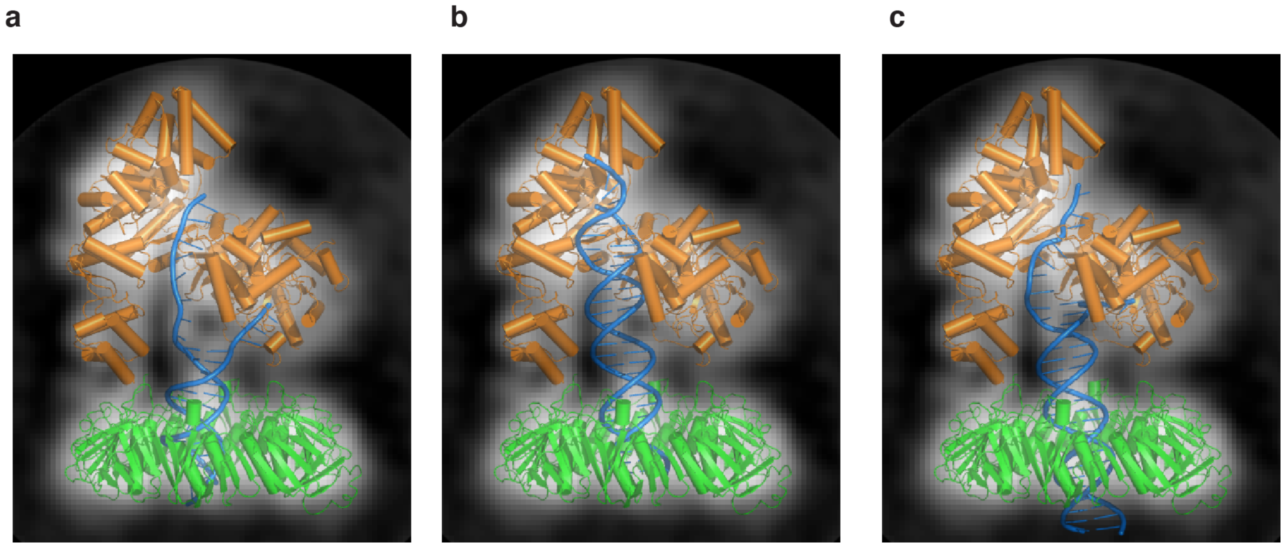

Supplementary Figure 4 | Modeling of the DNA into the 2D cryo-EM class. Different DNA models were fitted into the 2D cryo-EM class. (**a**) Model with the primer strand entering the PHP-exonuclease between short β-hairpin and long loop in the PHP domain (see Fig. 3). (**b**) DNA modeled in the polymerase mode (see Supplementary Fig. 1) (**c**) Model with the primer strand entering the PHP-exonuclease via the narrow groove between polymerase active site and exonuclease active site (see Fig. 2d).

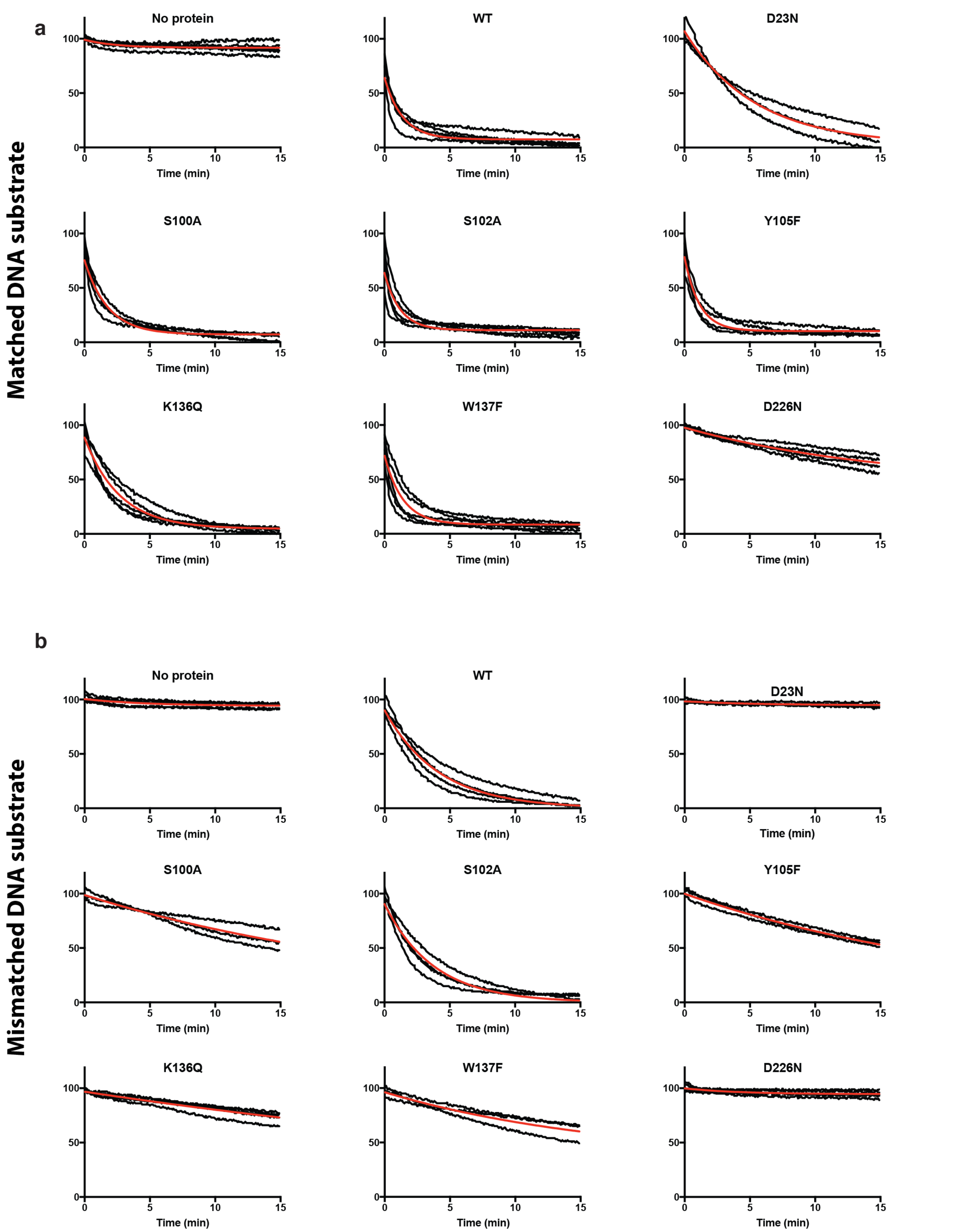

Supplementary Figure 5 | Real time polymerase assays on matched and mismatched DNA. Real-time primer extension assay in which the intensity of a 5' placed fluorophore is quenched by the incorporation of nucleotides in the bottom strand. (**a**) Primer extensions on a matched DNA substrate (**b**) Primer extension on mismatched DNA substrate. A single exponential decay was fitted to three to five independently measured experiments. Experimental curves in black, fitted curve in red.

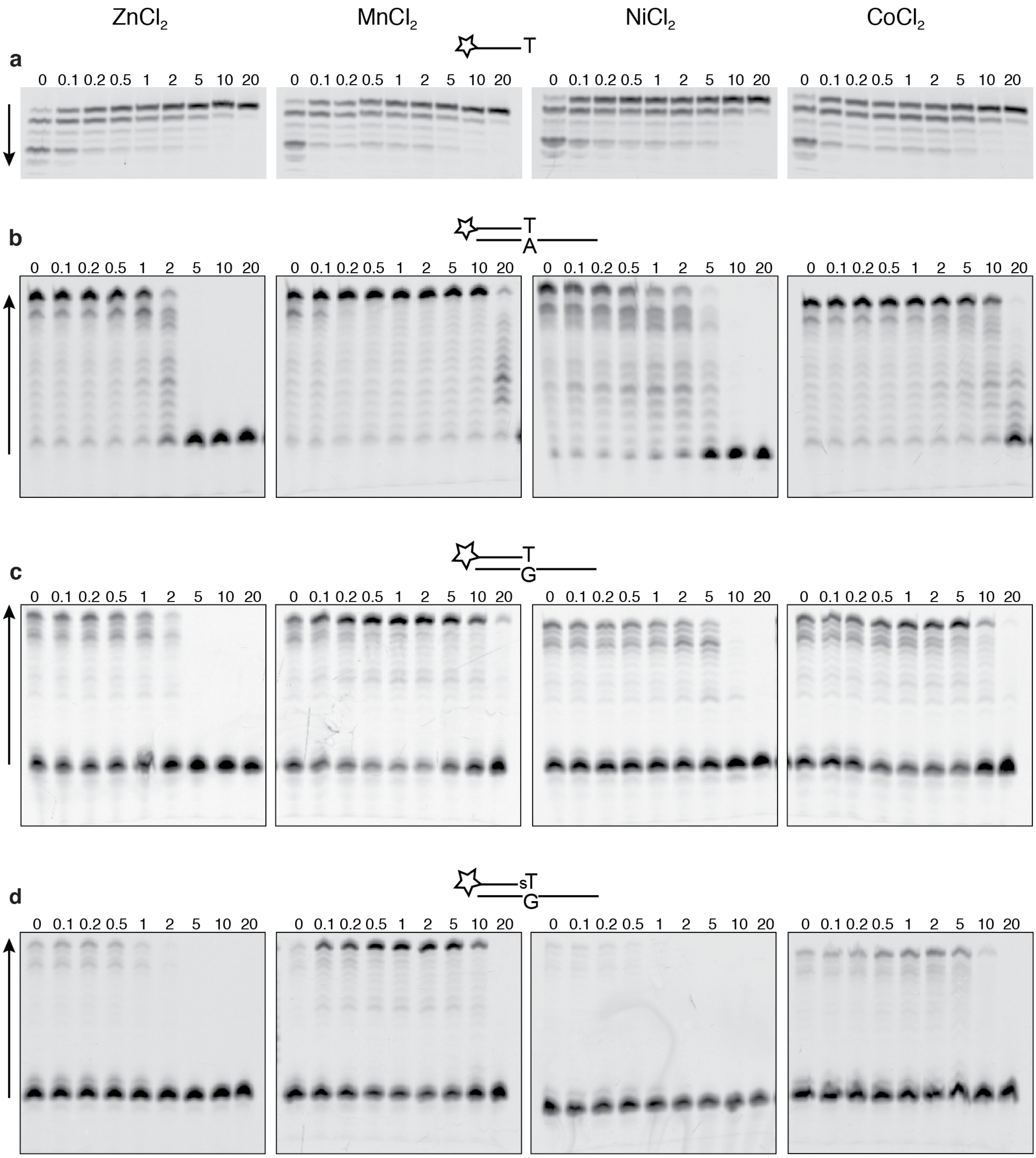

Supplementary Figure 6 | Exonuclease and polymerase inhibition by diverse metals. Different metals inhibit the PHP-exonuclease and polymerase at mM concentrations (**a**) Exonuclease activity on a single stranded DNA substrate (**b**) Polymerase activity on a matched substrate (**c**) Polymerase activity on a mismatched DNA substrate (**d**) Polymerase activity on a non-cleavable mismatched DNA substrate. Note that $MnCl_2$ enables the polymerase to extend from a mismatch, but inhibits the exonuclease. All reactions contained 2 mM Mg and increasing amounts of the indicated metal (0-20 mM). See main Fig. 4e-f for more details. Uncropped gels are shown in Supplementary Fig. 8.

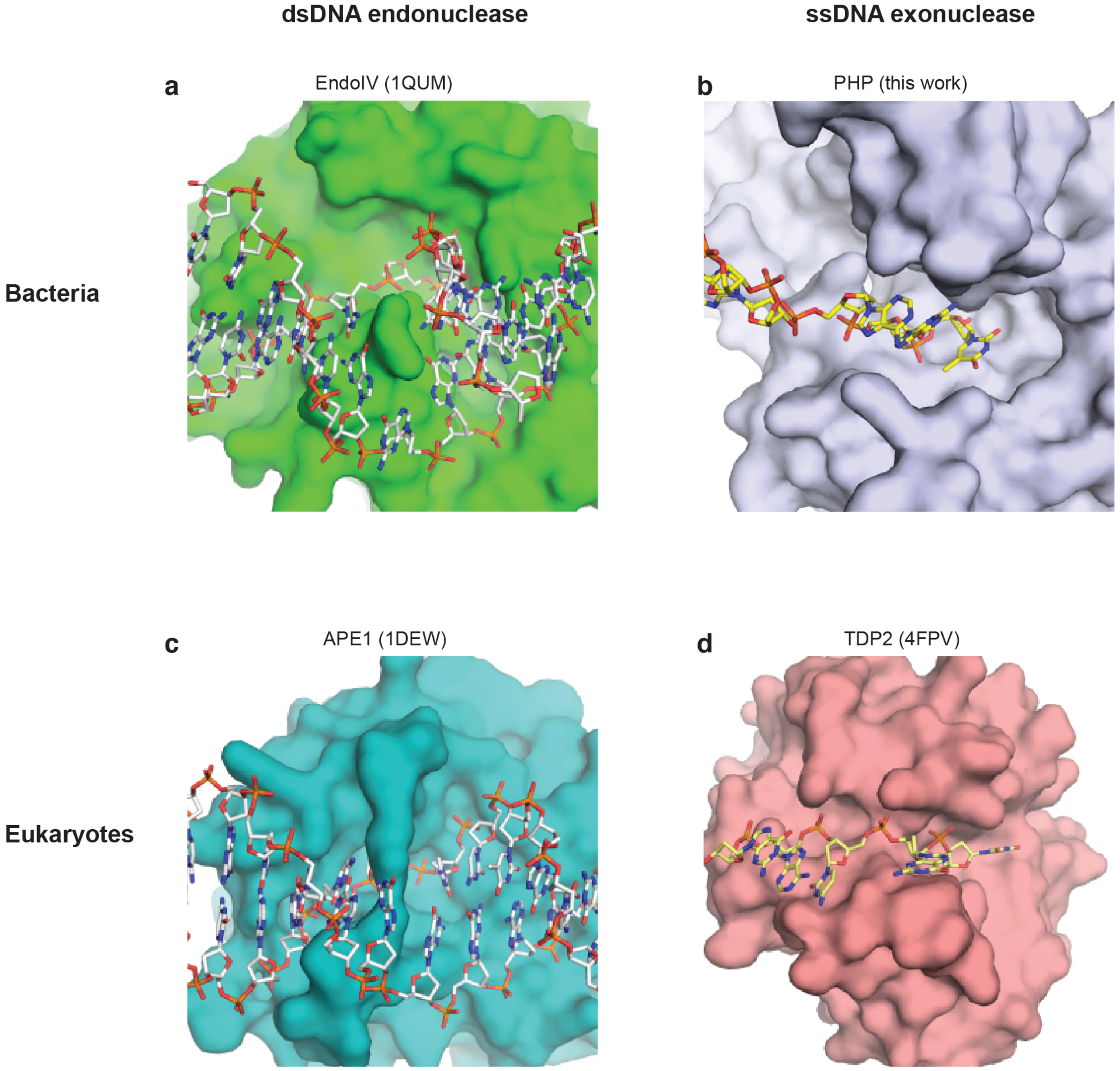

Supplementary Figure 7 | DNA binding in the endonuclease/exonuclease pairs EndoIV/PHP and APE1/TDP2 (**a**) dsDNA with an abasic site bound by *E. coli* Endo IV[11] (**b**) Modeling of ssDNA binding in PHP domain of Mtb DnaE1 (**c**) dsDNA with an abasic site bound by human APE1[12] (**d**) ssDNA binding in the zebrafish TDP2[13].

Supplementary References

1. Evans, R. J. *et al*. Structure of PolC reveals unique DNA binding and fidelity determinants. *Proc. Natl. Acad. Sci. USA* **105,** 20695–20700 (2008).
2. Fernandez-Leiro, R., Conrad, J., Scheres, S. H. & Lamers, M. H. cryo-EM structures of the E. coli replicative DNA polymerase reveal its dynamic interactions with the DNA sliding clamp, exonuclease and τ. *Elife* **4,** 459 (2015).
3. Bailey, S., Wing, R. A. & Steitz, T. A. The structure of T. aquaticus DNA polymerase III is distinct from eukaryotic replicative DNA polymerases. *Cell* **126,** 893–904 (2006).
4. Leulliot, N. *et al*. The family X DNA polymerase from Deinococcus radiodurans adopts a non-standard extended conformation. *J. Biol. Chem.* **284,** 11992–11999 (2009).
5. Lamers, M. H., Georgescu, R. E., Lee, S.-G., O'Donnell, M. & Kuriyan, J. Crystal structure of the catalytic alpha subunit of E. coli replicative DNA polymerase III. *Cell* **126,** 881–892 (2006).
6. Devos, J. M., Tomanicek, S. J., Jones, C. E., Nossal, N. G. & Mueser, T. C. Crystal structure of bacteriophage T4 5' nuclease in complex with a branched DNA reveals how flap endonuclease-1 family nucleases bind their substrates. *J. Biol. Chem.* **282,** 31713–31724 (2007).
7. Davies, D. R., Interthal, H., Champoux, J. J. & Hol, W. G. J. Explorations of peptide and oligonucleotide binding sites of tyrosyl-DNA phosphodiesterase using vanadate complexes. *J. Med. Chem.* **47,** 829–837 (2004).
8. Changela, A., DiGate, R. J. & Mondragón, A. Crystal structure of a complex of a type IA DNA topoisomerase with a single-stranded DNA molecule. *Nature* **411,** 1077–1081 (2001).
9. Lorentzen, E., Basquin, J., Tomecki, R., Dziembowski, A. & Conti, E. Structure of the active subunit of the yeast exosome core, Rrp44: diverse modes of substrate recruitment in the RNase II nuclease family. *Mol. Cell* **29,** 717–728 (2008).
10. Katoh, K. & Standley, D. M. MAFFT multiple sequence alignment software version 7: improvements in performance and usability. *Mol. Biol. Evol.* **30,** 772–780 (2013).
11. Hosfield, D. J., Guan, Y., Haas, B. J., Cunningham, R. P. & Tainer, J. A. Structure of the DNA repair enzyme endonuclease IV and its DNA complex: double-nucleotide flipping at abasic sites and three-metal-ion catalysis. *Cell* **98,** 397–408 (1999).
12. Mol, C. D., Izumi, T., Mitra, S. & Tainer, J. A. DNA-bound structures and mutants reveal abasic DNA binding by APE1 and DNA repair coordination [corrected]. *Nature* **403,** 451–456 (2000).
13. Shi, K. *et al*. Structural basis for recognition of 5'-phosphotyrosine adducts by Tdp2. *Nat. Struct. Mol. Biol.* **19,** 1372–1377 (2012).

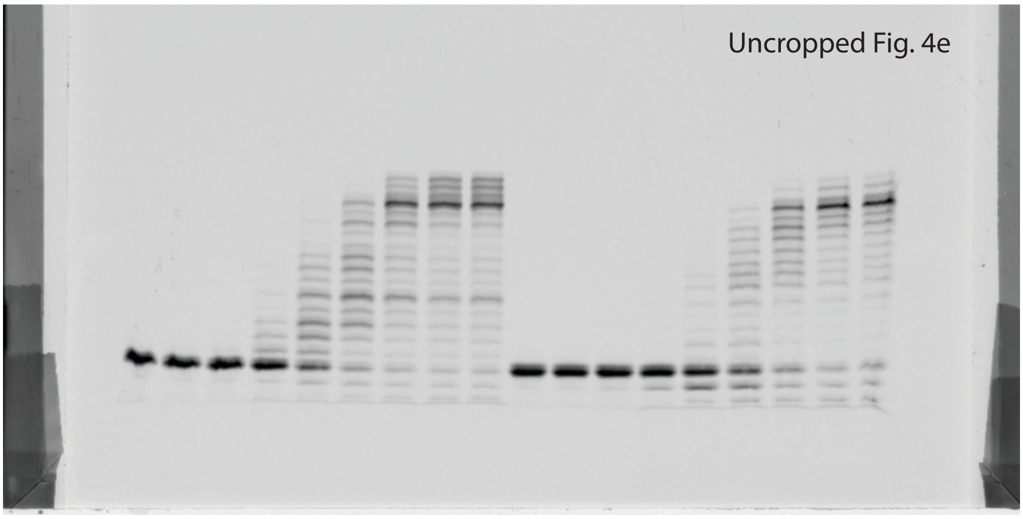

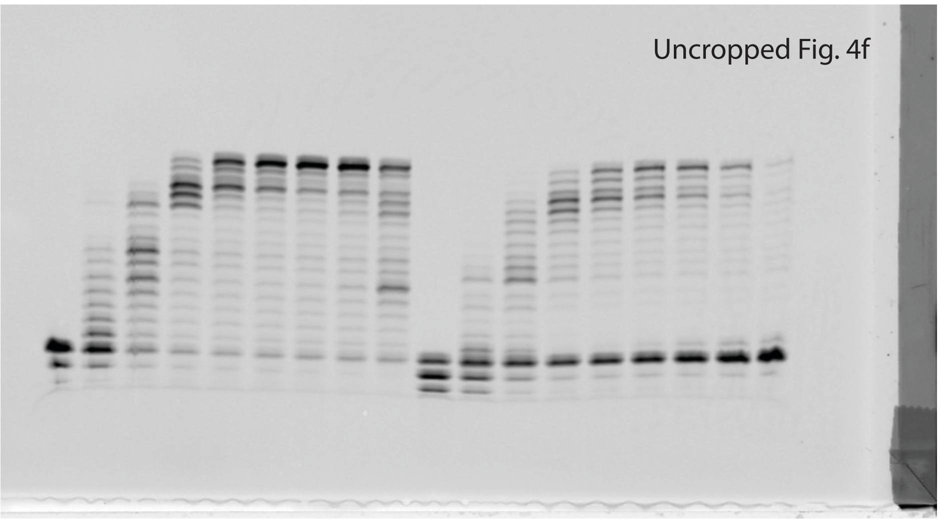

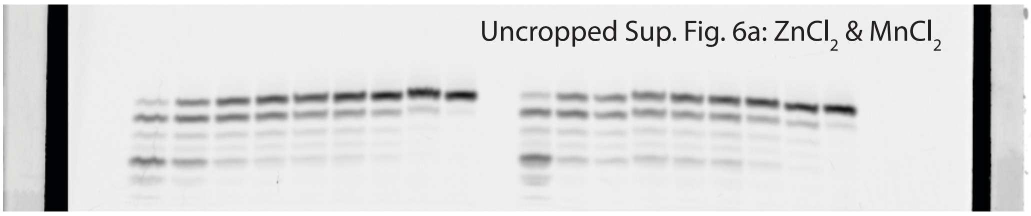

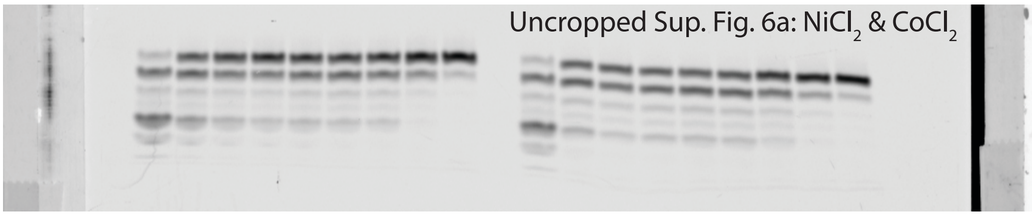

Supplementary Figure 8 | Uncropped gels for Figures 3e-f and Supplementary Figure 6a

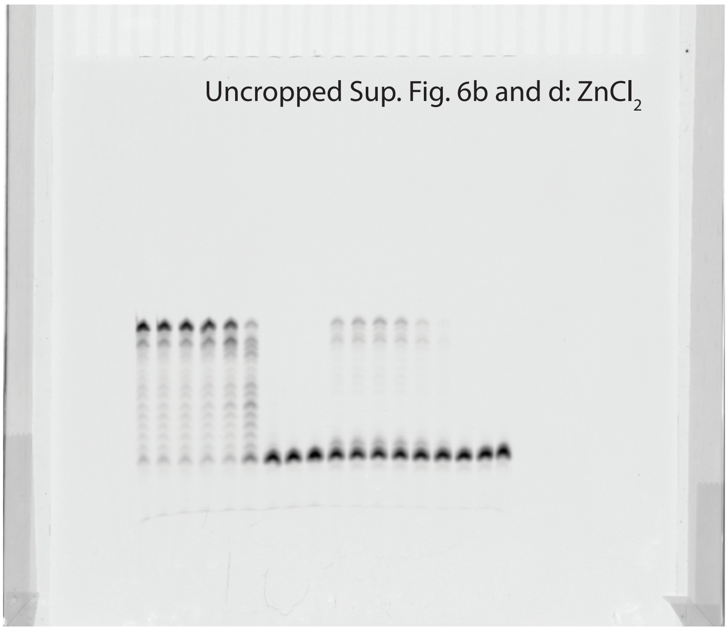

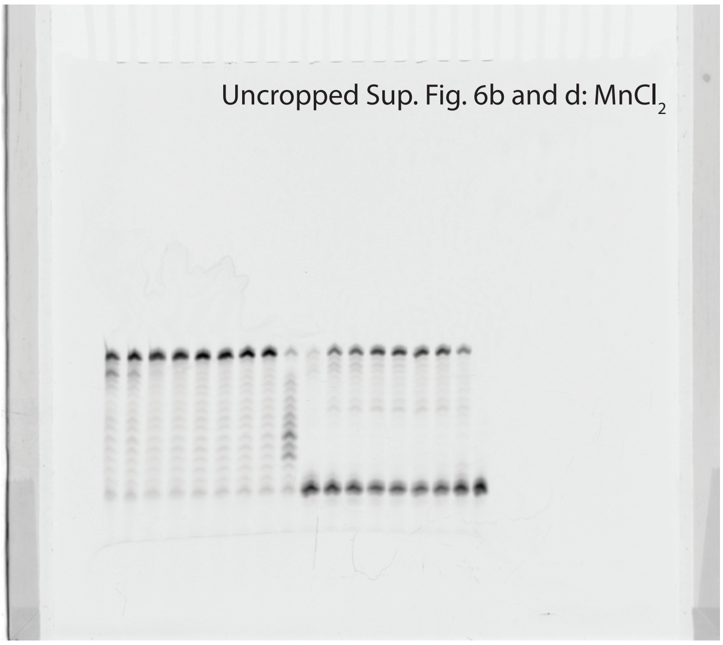

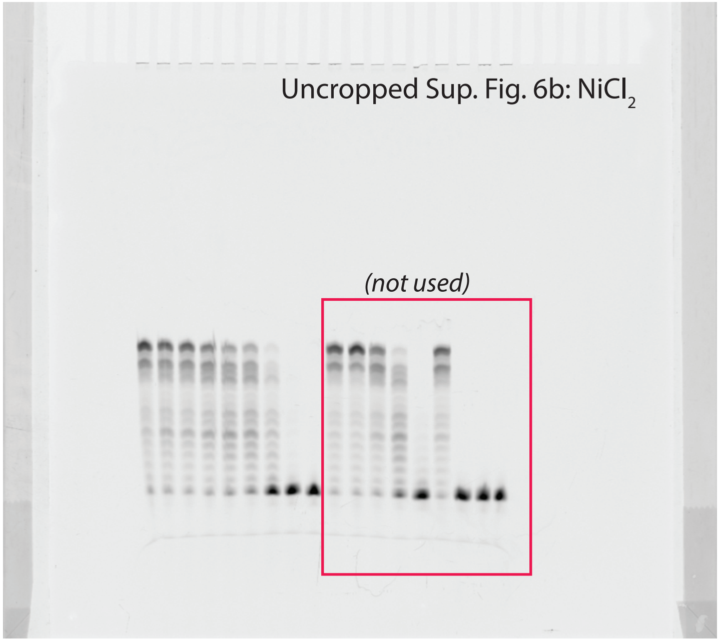

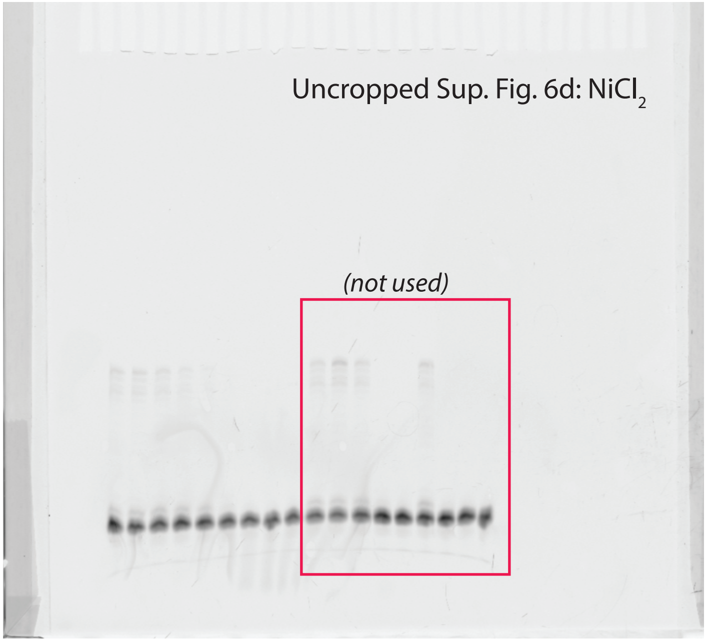

Supplementary Figure 8 (continued) | Uncropped gels for Supplementary Figure 6b and 6d

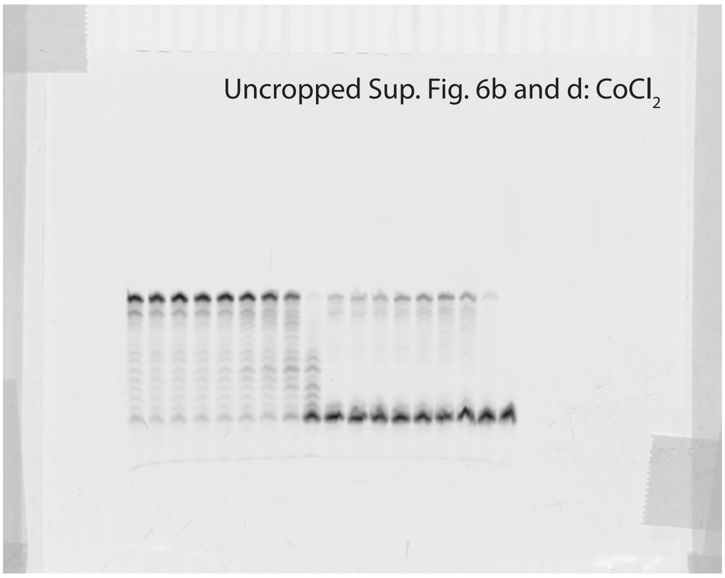

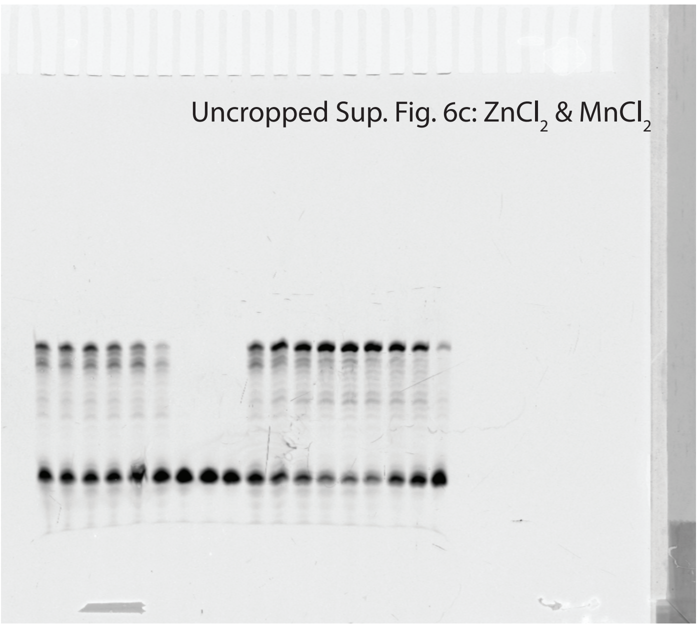

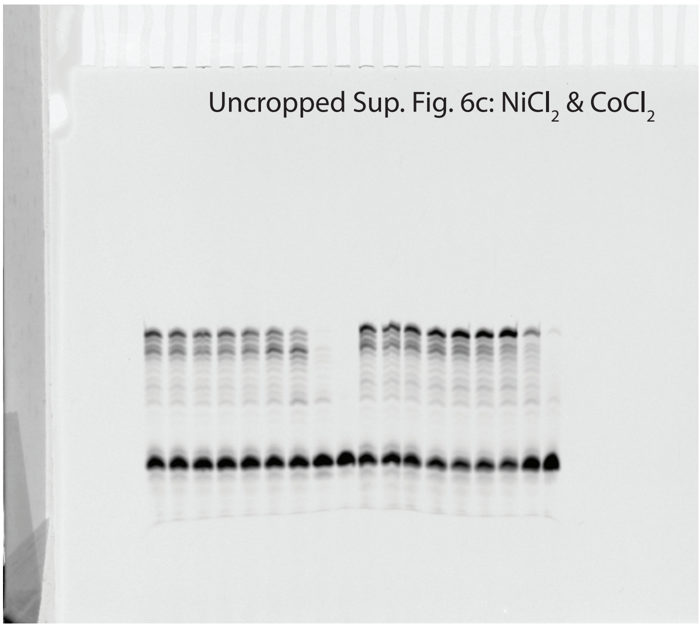

Supplementary Figure 8 (continued) | Uncropped gels for Supplementary Figure 6b-c
